# Brain endothelial homeostasis in psychiatric disorders: mechanisms, evidence, and translational opportunities

**DOI:** 10.3389/fncel.2026.1901256

**Published:** 2026-09-16

**Authors:** Mengjie Zhang, Xueying Huang, Lin Yao, Wenqing Wu

**Affiliations:** 1 Innovation Research Institute of Chinese Medicine, Shandong University of Traditional Chinese Medicine, Jinan, China; 2 Department of Pain Medicine, The First Affiliated Hospital of Shandong First Medical University & Shandong Provincial Qianfoshan Hospital, Jinan, China

**Keywords:** brain endothelium, CBF, depression, mental disorders, neurovascular coupling, psychiatric disorders

## Abstract

Brain endothelial cells (BECs) are specialized components of the neurovascular unit that couple circulating signals to cerebral blood flow regulation, blood–brain barrier (BBB) function, and selective molecular transport. This narrative review evaluates the correlation of these endothelial functions with psychiatric disorders, including depression and schizophrenia, distinguishing established vascular physiology from disease-specific evidence. In neurovascular coupling, the functions of capillary Kir2.1/phosphatidylinositol 4,5-bisphosphate and ATP-sensitive K^+^ channels, TRPA1-PANX1-P2X signaling, inositol 1,4,5-trisphosphate receptor-mediated Ca^2+^ events, arterial GJA4/GJA5 coupling, and PIEZO1-mediated mechanical feedback vary depending on the vascular segment. At the BBB, CLDN5, OCLN, F11R, TJP1, CDH5, the actin cytoskeleton, MFSD2A, and selective influx and efflux transporters determine paracellular permeability and transcytosis. Cerebral fluid and solute clearance should be separated into perivascular cerebrospinal fluid/interstitial fluid exchange, BBB transport, and meningeal lymphatic or venous drainage. BECs contribute to vascular and transporter interfaces but are not demonstrated to be the central drivers of glymphatic flow. Stress-related, sex-dependent, and brain region-dependent changes in CLDN5 and endothelial inflammatory signaling are reported to be strongly correlated with psychiatric disorders. In contrast, studies on serum biomarkers, postmortem tissues, patient-derived models, and animals provide different and non-equivalent levels of inference. Clinical trials have not evaluated a BEC-directed therapy for a primary psychiatric disorder. Future studies must focus on developing endothelial-specific and vascular segment-specific interventions, identifying prospective human BBB or cerebrovascular biomarkers, performing analysis according to sex and brain region, and systematically assessing peripheral vascular and barrier-related safety.

## 1 Introduction

Psychiatric disorders, which are major contributors to global disability, encompass heterogeneous alterations in cognition, emotion, behavior, and social functioning (GBD 2019 Mental Disorders Collaborators, 2022). The pathophysiology of psychiatric disorders cannot be attributed to isolated neuronal aberrations. Neural activity, glial signaling, immune responses, metabolism, and vascular function interact within the neurovascular unit (NVU). This provides a framework for examining the regulatory effects of systemic and cerebral processes on psychiatric phenotypes (Iadecola, 2017; McConnell et al., 2019; Sullivan et al., 2024). In this framework, vascular aberrations may function as contributors or modifiers or may be the consequences of disease rather than serving as a single universal cause (Figure 1).

**Figure 1:**
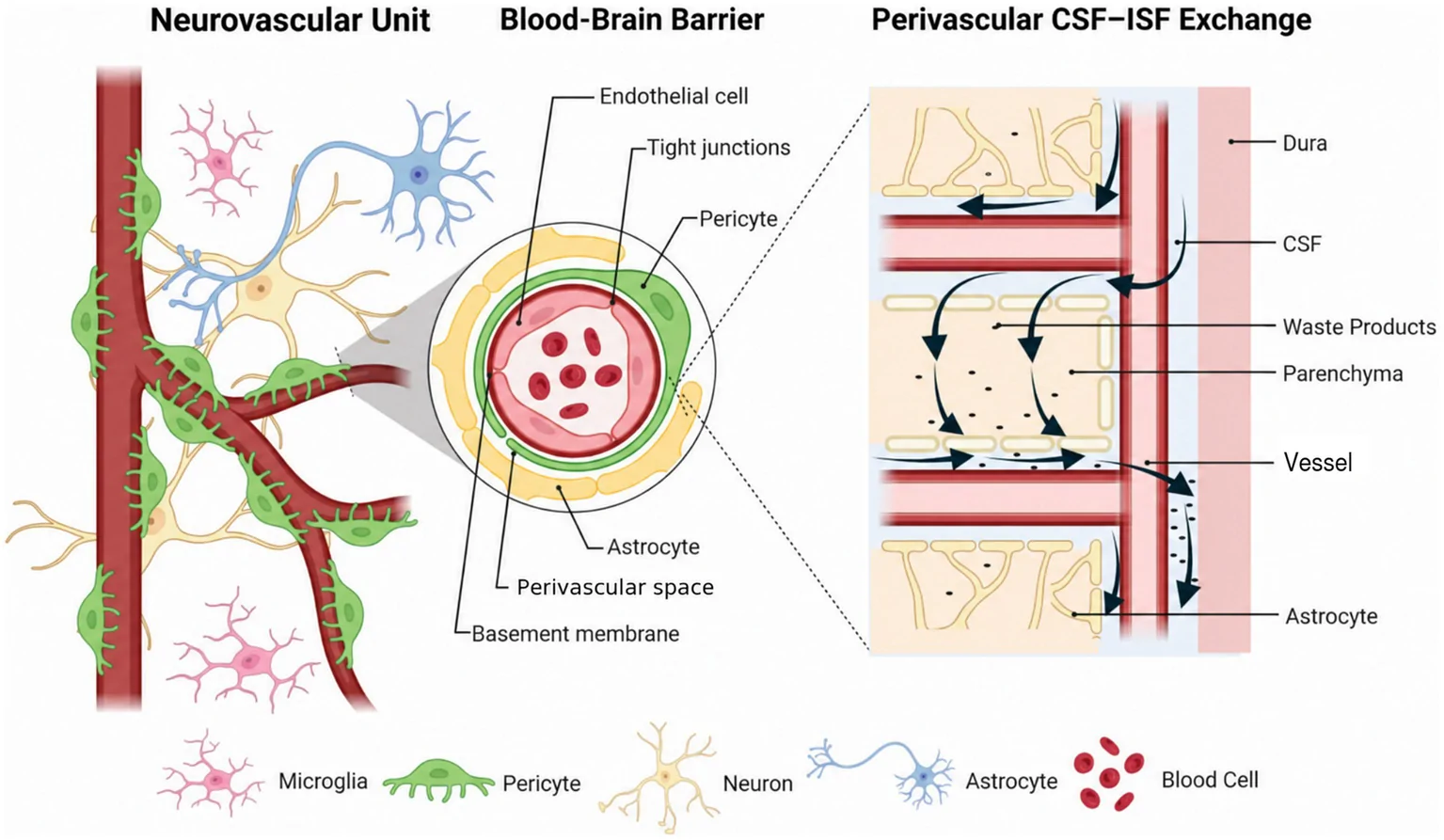
Structural organization of the neurovascular unit, blood–brain barrier, and cerebral fluid clearance interfaces. The left panel shows brain endothelial cells (BECs) integrated with pericytes, astrocytic endfeet, neurons, microglia, basement membranes, and circulating blood cells. The enlarged vessel cross-section emphasizes the endothelial monolayer, tight junctions, pericyte coverage, basement membranes, and the perivascular compartment enclosed by astrocytic endfeet. The right panel schematically distinguishes cerebrospinal fluid movement from subarachnoid/periarterial spaces, exchange with interstitial fluid in the parenchyma, and downstream movement toward perivenous and meningeal drainage routes. Astrocytic endfeet, especially polarized AQP4, support water exchange. BECs regulate vascular and transporter interfaces but are not portrayed as the sole driver of glymphatic flow. Arrows indicate proposed bulk-flow or exchange directions rather than direct endothelial transport.

Brain endothelial cells (BECs), which are the luminal cellular layer of cerebral vessels, acquire barrier and signaling properties through interactions with pericytes, astrocytic endfeet, vascular smooth muscle cells, basement membranes, neurons, and immune cells (Abbott et al., 2006; Lu et al., 2023; Zhao et al., 2022; Bayir and Sendemir, 2021). Additionally, BECs contribute to neurovascular coupling (NVC), establish the principal cellular interface of the blood–brain barrier (BBB), and mediate substrate-specific influx and efflux. BECs exhibit an indirect correlation with perivascular cerebrospinal fluid (CSF) exchange, which must be distinguished from astrocytic water transport and meningeal lymphatic drainage. Analysis of clinical biomarkers, postmortem tissues, patient-derived models, and stress paradigms revealed the occurrence of endothelial aberrations in depression, schizophrenia, and related disorders. However, these evidence sources differ in anatomical specificity and causal strength (Huppert et al., 2010; Choi et al., 2020; Buzhdygan et al., 2024; Chen et al., 2025). This review examined segment-specific endothelial mechanisms in cerebral blood flow (CBF) regulation, molecular modulation of BBB integrity, cerebral fluid and solute clearance pathways, sex-dependent and brain region-dependent responses, and the evidence hierarchy supporting BEC-directed therapeutic strategies. The major endothelial functions, molecular pathways, and evidence levels discussed in this review have been summarized in Table 1.

**Table 1:** Summary of the associations between BEC homeostasis dysregulation and psychiatric disorders.

Type of psychiatric disorder	Pathological mechanisms	Evidence source/Model	Key molecules/Pathways	Major pathological effects	References
Depression	Neurovascular uncoupling	Adult mouse chronic-stress models	eNOS	Reduced nitric oxide production, impaired vasodilation, and disrupted cerebral blood-flow regulation.	Zhang et al. (2024)
Depression	Neurovascular uncoupling	Separate high-salt mouse models	ROCK	IL-17A-associated ROCK activation suppresses eNOS signaling and contributes to cerebrovascular endothelial dysfunction.	Faraco et al. (2018) and Lu et al. (2025)
Depression	Neurovascular uncoupling	Endothelial-specific developmental knockout mouse	GABAA receptor β3 subunit	Developmental endothelial deletion produces microvascular abnormalities, altered cerebral blood flow, and behavioral dysfunction.	Agrud et al. (2022)
Depression	Intercellular inflammatory interactions	Review and mouse stress model	CCL2; IL-1β	Stress-related microglial signaling recruits inflammatory monocytes to brain endothelium and promotes anxiety- and depression-related behavior.	Weber et al. (2017) and McKim et al. (2018)
Depression	BBB disruption	Human postmortem tissue and mouse stress models	CLDN5	Region- and sex-dependent reductions in CLDN5 are associated with focal BBB permeability and stress susceptibility.	Dudek et al. (2020) and Menard et al. (2017) and Dion-Albert et al. (2022)
Schizophrenia	Neurovascular uncoupling	Cell and experimental vascular models	Endothelial NMDAR	Impaired endothelial NMDAR signaling may weaken astrocyte-evoked nitric oxide generation and local vasodilation in experimental systems.	Lu et al. (2019)
Schizophrenia	BBB-associated abnormalities	Human serum and patient-derived models	CLDN5; angiogenesis- and permeability-related pathways	Serum, organoid, and patient-derived cellular studies indicate barrier-associated abnormalities, although causal inference remains limited.	Usta et al. (2021) and Stankovic et al. (2024) and Lizano et al. (2023)
Bipolar disorder	BBB-associated abnormalities	Human serum and patient-derived evidence	CLDN5; endothelial permeability pathways	Serum and patient-derived cellular evidence suggests barrier-related abnormalities without establishing brain-endothelial causality.	Kılıç et al. (2020) and Lizano et al. (2023)
Anxiety-related phenotypes	Neurovascular and inflammatory interactions	Developmental knockout and mouse stress models	GABAA receptor β3 subunit; CCL2; IL-1β	Vascular developmental abnormalities and stress-related monocyte recruitment contribute to anxiety-like behavior in animal models.	Agrud et al. (2022) and Weber et al. (2017) and McKim et al. (2018)
Cocaine use disorder-associated psychiatric abnormalities	Neurovascular dysfunction	Human observational study	sICAM-1; RANTES	Circulating endothelial activation markers correlate with regional cerebral blood-flow abnormalities, without proving a brain-endothelial causal mechanism.	Massardo et al. (2015)

Evidence sources are heterogeneous and include human observational studies, postmortem tissue, patient-derived models, and animal experiments; the table does not imply equivalent causal strength across disorders or mechanisms.

## 2 Brain endothelial contributions to NVC

### 2.1 Segment-specific endothelial mechanisms regulating CBF

NVC is a multicellular process involving neurons, astrocytes, pericytes, vascular smooth muscle cells, and endothelial cells. BECs are associated with segment-specific sensing and signal conduction functions along capillaries, transitional vessels, arterioles, arteries, and veins rather than functioning as a single dominant regulator. Capillary BECs detect changes in extracellular ions, metabolites, and membrane potential, whereas arterial endothelial cells are involved in shaping vasomotor responses. These coordinated mechanisms match local CBF to neuronal metabolic demand (Andreone et al., 2015).

#### 2.1.1 Capillary potassium-sensitive and metabolite-sensitive electrical signaling

Increased neuronal activity enhances the levels of extracellular K^+^, which activates the inwardly rectifying potassium channel Kir2.1 in capillary endothelial cells and initiates retrograde hyperpolarization toward upstream arterioles (Negri et al., 2021). Kir2.1 activity is dependent on phosphatidylinositol 4,5-bisphosphate (PIP2). The depletion of endothelial PIP2 attenuates capillary electrical signaling (Harraz et al., 2018; Longden et al., 2017). Kir6.1-containing ATP-sensitive potassium (KATP) channels in both capillary endothelial cells and pericytes are involved in a separate metabolic pathway. Adenosine activates KATP channels through A2A receptors and the Gαs/cAMP/protein kinase A pathway, increasing CBF (Sancho et al., 2022). In parenchymal arterioles, endothelial small-conductance and intermediate-conductance Ca^2+^-activated K^+^ channels (SKCa and IKCa, respectively) contribute to endothelial hyperpolarization and vasodilation. These endothelial effects do not exclude an important role for large-conductance calcium-activated and voltage-activated potassium (BKCa) channels, which exert their effects in a different cellular compartment, in vascular smooth muscle (Hannah et al., 2011).

#### 2.1.2 Flow-dependent PIEZO1 feedback

PIEZO1 is a mechanically activated cation channel expressed in the capillary endothelium of the central nervous system (CNS) (Harraz et al., 2022; Lan et al., 2024). Recent studies have revealed that endothelial PIEZO1 functions primarily as a feedback sensor of the hemodynamic forces generated after functional hyperemia initiation in the brain. PIEZO1-mediated inward current depolarizes endothelial cells, opposes sustained hyperpolarization, and promotes the return of blood flow toward baseline (Lim et al., 2024). Additionally, PIEZO1 can couple to Ca^2+^-related and nitric oxide (NO)-related pathways in other vascular contexts but should not be interpreted as an established feed-forward initiator of neurovascular vasodilation in brain capillaries.

#### 2.1.3 Calcium-sensitive and redox-sensitive endothelial signaling

Capillary endothelial Ca^2+^ signals are spatially heterogeneous. Local and propagating inositol 1,4,5-trisphosphate receptor (IP3R)-mediated Ca^2+^ events can summate and influence downstream vascular responses (Longden et al., 2021). Endothelial TRPA1 channels are associated with another experimentally defined pathway. In this pathway, 4-hydroxynonenal (4-HNE) activates TRPA1, leading to PANX1-dependent ATP release, P2X receptor activation in transitional vascular segments, and conversion of a relatively slow Ca^2+^ signal into a rapid electrical signal that reaches upstream arterioles (Thakore et al., 2021). However, 4-HNE was identified as an effective experimental TRPA1 agonist. The amount, source, and timing of 4-HNE during physiological neuronal activation have not been determined. Recent studies on electrocalcium coupling indicated that capillary hyperpolarization increases the electrochemical driving force for Ca^2+^ entry and consequently activates local Ca^2+^ signals (Mughal et al., 2024). This finding should be distinguished from previous claims that Kir2.1 directly triggers IP3R-dependent Ca^2+^ release from the endoplasmic reticulum.

#### 2.1.4 Endothelial gap junction conduction and vascular zonation

Endothelial-endothelial gap junctions provide a distinct route for rapid, long-range propagation of vasodilatory signals. In awake mice, Gja4 and Gja5 are enriched in arterial endothelial cells. Acute endothelial deletion of *Gja4* and *Gja5* impaired both the speed and spatial extent of neurally evoked vasodilation (Krolak et al., 2025). This arterial endothelial coupling is distinct from myoendothelial communication between endothelial and smooth muscle cells. Previous studies have established vascular segment-specific physiology. However, the role of GJA4/GJA5 dysfunction in psychiatric disease and the value of GJA4/GJA5 as a tractable psychiatric therapeutic target have not been examined.

### 2.2 Evidence linking endothelial dysregulation to psychiatric phenotypes

Previous studies have reported altered cerebral perfusion and endothelial aberrations in several psychiatric conditions. However, the strength and direction of evidence vary across human observational studies, cell and tissue preparations, and animal models. Human studies have reported predominantly associative data and do not establish BEC dysfunction as an initiating cause of psychiatric symptoms. Mechanistic claims require explicit separation of clinical associations from endothelial-specific experimental interventions.

#### 2.2.1 Human and ex vivo evidence

One study reported that chronic stress exposure and stress appraisal are associated with impaired peripheral endothelial function in young adults. However, this study did not directly examine cerebral endothelium or NVC (Carlson et al., 1985). In cocaine-dependent individuals, regional CBF changes are correlated with circulating soluble ICAM1 and CCL5. However, the cellular origin of ICAM1 and CCL5 and a causal role for BECs were not demonstrated (Massardo et al., 2015). Endothelial N-methyl-D-aspartate receptors (NMDARs) can potentially contribute to cortical vasodilatory signaling. Primary BECs express GRIN1. The silencing of endothelial *GRIN1* attenuates astrocyte-evoked NO generation and local vasodilation in experimental preparations (Lu et al., 2019). However, cerebral hypoperfusion in schizophrenia cannot be attributed specifically to endothelial NMDAR dysfunction based on these data. Similarly, Na^+^/K^+^-ATPase inhibition promoted Ca^2+^ release from the endoplasmic reticulum, Gsk3b activation, and endothelial degeneration in rat prefrontal cortex slice cultures. However, this study did not examine NVC, cerebral perfusion, or psychiatric behavior (Kurauchi et al., 2016).

#### 2.2.2 Evidence from animal models

Chronic unpredictable mild stress and chronic social defeat stress, but not maternal separation, are associated with microvascular dysfunction before the emergence of depression-like behavior. Nos3^+/−^ mice developed microvascular impairment, which was followed by behavioral aberrations. Improving NO-dependent vascular function ameliorated the behavioral aberrations (Zhang et al., 2024). These findings support a vascular contribution in specific adult stress models but should not be generalized to all depressive disorders. High dietary salt suppressed TH17/IL17A-dependent endothelial NO signaling and neurovascular dysfunction in one model. Meanwhile, one study reported that high salt and IL17A were correlated with depression-like behavior (Faraco et al., 2018; Lu et al., 2025). These studies suggest convergent biology but do not demonstrate a single continuous IL-17A-ROCK-eNOS-depression pathway. Endothelial-specific developmental deletion of *Gabrb3* resulted in reduced vascular density, increased cortical capillary blood flow, and broad behavioral aberrations. The manipulation was introduced during development and affected systemic vasculature. Hence, this manipulation is not equivalent to acquired adult endothelial dysfunction in depression or schizophrenia (Agrud et al., 2022). Repeated social defeat stress promotes the microglial CCL2-dependent recruitment of inflammatory monocytes to brain endothelium, where monocyte-derived IL1B contributes to anxiety-like behavior (Weber et al., 2017; McKim et al., 2018). This mechanism is more specific than attributing the effect broadly to vascular endothelial growth factor (VEGF) or astrocytic endfeet loss, which was not supported by the cited studies.

#### 2.2.3 Therapeutic implications and current limitations

The endothelial pathways described above identify control points that can be experimentally manipulated. However, these pathways have not been validated as a BEC-directed treatment for a primary psychiatric disorder. PIP2/Kir2.1 and A2A-KATP signaling can enhance capillary electrical signaling, while GJA4/GJA5 coupling determines arterial signal propagation. Conversely, PIEZO1 functions as a feedback brake, rendering simple agonist-versus-antagonist strategies biologically inappropriate (Harraz et al., 2018; Sancho et al., 2022; Lim et al., 2024; Krolak et al., 2025). Therapeutic strategies must be selective for BECs and vascular segments, demonstrated to be effective in disease-relevant models, and exert regulatory effects on systemic blood pressure and perfusion (Figure 2).

**Figure 2:**
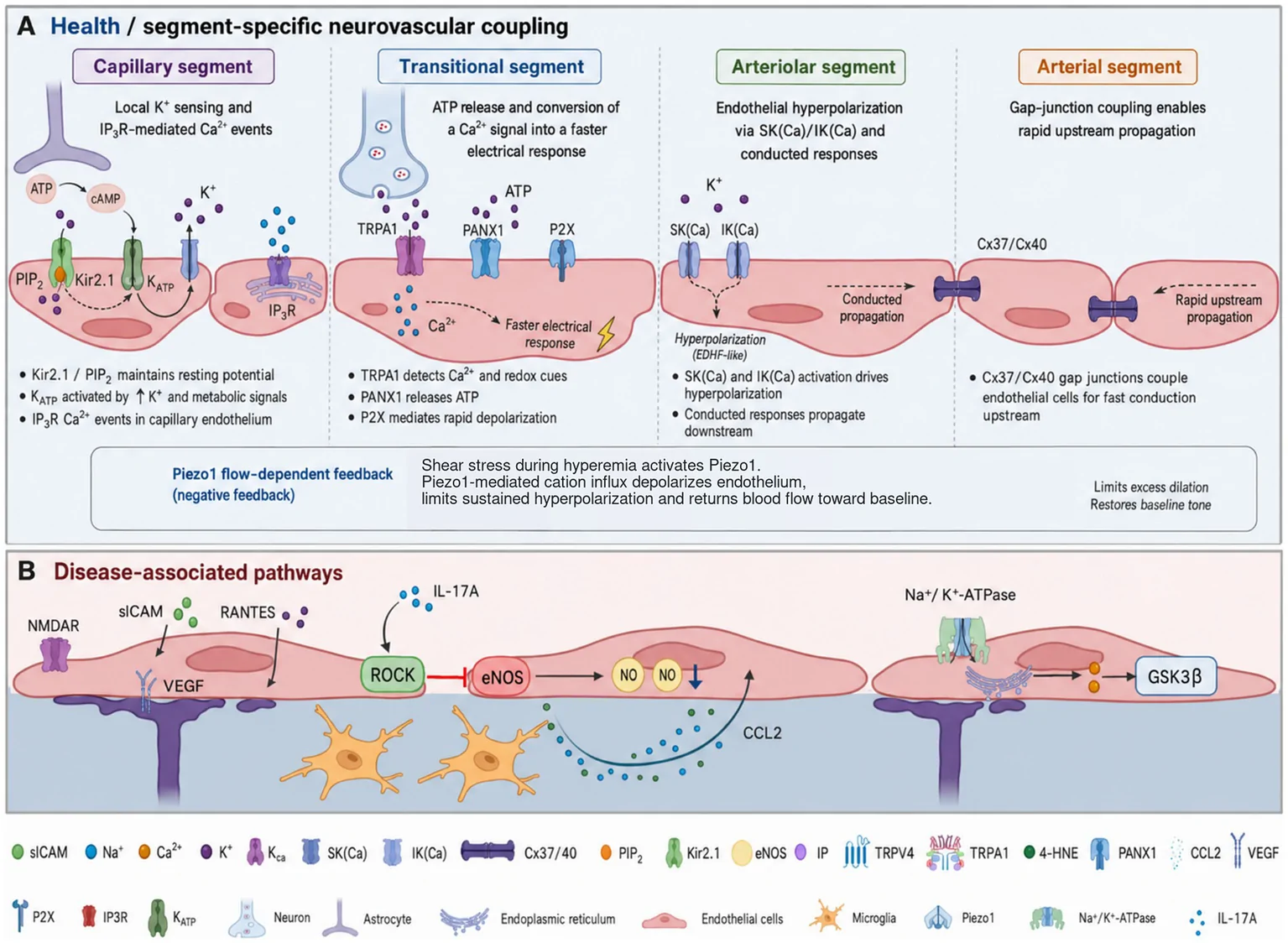
Correlation of segment-specific endothelial mechanisms in neurovascular coupling with psychiatric phenotypes. Upper panel: Capillary K^+^ sensing through Kir2.1/phosphatidylinositol 4,5-bisphosphate (PIP_2_) and ATP-sensitive K^+^ channels, endothelial small-conductance/intermediate-conductance Ca^2+^-activated K^+^ channel (SKCa/IKCa) activities, inositol 1,4,5-trisphosphate receptor (IP_3_R)-mediated Ca^2+^ signaling, TRPA1-PANX1-P2X signaling, GJA4/GJA5-dependent conducted responses, and PIEZO1-mediated mechanical feedback are shown according to vascular segment. Purple, orange, and blue symbols denote K^+^, Ca^2+^, and Na^+^, respectively. Lower panel: Proposed disease-associated pathways include N-methyl-D-aspartate receptor (NMDAR) signaling, IL-17A-ROCK-eNOS suppression, altered Na^+^/K^+^-ATPase-GSK3β signaling, endothelial adhesion markers, CCL2 release, and microglial/endothelial inflammatory interactions. The disease panel integrates cell and animal evidence and should not be interpreted as proof that every pathway is causal or clinically targetable in psychiatric disorders. In capillary endothelial cells, Kir2.1-dependent and KATP-dependent hyperpolarization links extracellular K^+^ and adenosine signaling to upstream vasodilation. In contrast, TRPA1/IP_3_R-related Ca^2+^ signals are converted into rapid electrical responses at transitional vascular segments. In the arterial endothelium, GJA4/GJA5 gap junctions support the rapid longitudinal propagation of vasodilation. PIEZO1 should be interpreted as a flow-dependent negative-feedback sensor that facilitates recovery from hyperemia rather than as an initiating vasodilator. The lower panel summarizes proposed disease-related mechanisms from heterogeneous experimental systems. Na^+^/K^+^-ATPase inhibition and GSK3β activation are based on ex vivo endothelial degeneration, whereas stress-related, Il17a-related, Gabrb3-related, and Ccl2-related pathways are derived from distinct animal models and should not be interpreted as one continuous mechanism or as established causal factors in humans.

## 3 Brain endothelial regulation of BBB integrity

### 3.1 Molecular and cellular basis of the brain endothelial barrier

The BBB is a multicellular neurovascular interface rather than an endothelial layer acting in isolation. BECs form the luminal barrier, while pericytes, astrocytic endfeet, vascular smooth muscle cells in arteriolar segments, basement membranes, and extracellular matrix components regulate the development and maintenance of the BBB. BECs restrict paracellular and nonspecific transcellular passage, provide receptor-mediated and carrier-mediated transport, and exchange signals with neighboring mural and glial cells. These properties vary across capillary, venous, and arterial segments and can change with development, inflammation, and disease (Bazzoni and Dejana, 2004; Alahmari, 2021; Daneman and Prat, 2015).

#### 3.1.1 Intercellular junctions and cytoskeletal anchoring maintain BBB integrity

Continuous tight junctions between adjacent BECs restrict paracellular diffusion. CLDN5, which is the predominant endothelial claudin, forms a size-selective seal. The genetic loss of *Cldn5* in mice preferentially increases permeability to small molecules without abolishing vascular morphology (Greene et al., 2019; Nitta et al., 2003). OCLN is associated with claudin-based strands and contributes to junctional organization and signaling. However, the presence of OCLN alone does not determine barrier tightness. F11R is involved in cell–cell adhesion, leukocyte-endothelial interactions, and junction assembly. Experimental BBB breakdown is associated with the redistribution or downregulation of F11R (Yeung et al., 2008). On the cytoplasmic side, zonula occludens proteins, especially TJP1, link claudins and OCLN to the perijunctional actin cytoskeleton and stabilize the solute barrier (Van Itallie et al., 2009). Adherens junctions are organized primarily around CDH5, which binds catenins and actin. Therefore, CDH5 is not merely a parallel adhesive structure. CDH5 promotes *CLDN5* transcription and functionally couples adherens-junction integrity to tight junction maintenance through AKT-FoxO1-beta-catenin signaling (Taddei et al., 2008). Inflammation, oxidative stress, matrix metalloproteinases, or cytoskeletal contraction may disrupt different components of this complex. Changes in a circulating junctional marker should not be interpreted as direct evidence of structural BBB leakage.

#### 3.1.2 Selective transport and suppression of transcytosis regulate substance exchange

BECs combine selective influx, regulated efflux, and low rates of vesicular transcytosis (Jhaveri et al., 2024). GLUT1 supplies glucose, amino-acid carriers support nutrient exchange, and ion transporters maintain the ionic environment required for neuronal function (Alahmari, 2021; Daneman and Prat, 2015; Dyatlova et al., 2022; Won et al., 2015; Dietschy and Turley, 2001; Mitchell et al., 2011). MFSD2A is upregulated in the endothelium of the CNS, transports lysophosphatidylcholine-bound lipids, and suppresses caveolae-mediated transcytosis. The loss of *Mfsd2a* in mice promotes BBB leakage despite preserved tight junction morphology (Ben-Zvi et al., 2014). ATP-binding cassette transporters, including P-glycoprotein, limit entry or promote the removal of defined substrates. These systems are molecularly selective and should not be described as a single nonspecific pathway for nutrient entry or waste clearance.

#### 3.1.3 Interactions with pericytes, astrocytes, and basement membranes

Barrier properties are dependent on reciprocal signaling within the NVU. During vascular development, endothelial PDGFB recruits PDGFRB-expressing pericytes. Pericyte coverage limits endothelial transcytosis, supports basement-membrane organization, and maintains endothelial gene expression (Hellström et al., 1999; Kemp et al., 2020; Mäe et al., 2021; Gautam et al., 2016). Endothelial-pericyte signaling is context-dependent. Factors, such as TGFB, sphingosine-1-phosphate, angiopoietins, and VEGF, can stabilize or destabilize the barrier according to concentration, receptor distribution, and disease state. Thus, VEGF should not be presented as a uniformly protective factor for junctions (Daneman and Prat, 2015; Huang, 2020; Lin et al., 2025). Astrocytic endfeet contribute extracellular matrix and trophic signals and regulate water and ion homeostasis. However, most mechanistic evidence for astrocytic endfeet is derived from developmental models or reductionist co-culture systems. Astrocytic GJA1 signaling and endothelial junctional integrity may interact during inflammation. However, this interaction does not establish a universal linear pathway from astrocytic calcium signaling to BEC permeability (Garcia et al., 2004; Gautam et al., 2019; Boulay et al., 2015; De Bock et al., 2022). Thus, the BBB is maintained by coordinated cellular and matrix interactions, with BECs providing the physical interface without functioning as the sole regulator.

### 3.2 Evidence linking BBB alterations to psychiatric disorders

BBB-related aberrations have been reported in depression, schizophrenia, bipolar disorder, obsessive-compulsive disorder, and other psychiatric conditions. However, the underlying evidence is heterogeneous (Usta et al., 2021; Fiorentino et al., 2016; Kealy et al., 2020; Sălcudean et al., 2025; Işık et al., 2020; Kılıç et al., 2020; Dudek et al., 2020; Menard et al., 2017; Dion-Albert et al., 2022; Stankovic et al., 2024; Lizano et al., 2023). Human studies have focused on serum biomarkers, postmortem tissues, neuroimaging, patient-derived cells, and organoids. Each human study captured a different aspect of vascular biology, which is vulnerable to confounding factors, such as medication exposure, smoking, metabolic disease, and agonal state. Animal and cell models can examine mechanisms but cannot establish their causal roles in human psychiatric disorders. Therefore, the evidence supports disease association and biological plausibility more strongly than a general causal claim.

The most developed mechanistic evidence is stress-related phenotypes and CLDN5. In male mice exposed to chronic social defeat stress, the loss of endothelial *Cldn5* in the nucleus accumbens is associated with focal BBB permeability, entry of circulating IL6, and depression-like behavior. Human postmortem nucleus accumbens samples from patients with major depressive disorder exhibited CLDN5 downregulation. However, the temporal expression patterns of CLDN5 have not been determined in humans (Menard et al., 2017). A subsequent study combined human postmortem observations with mouse experiments and demonstrated that endothelial inflammatory and epigenetic programs differ between stress-susceptible and stress-resilient animals. Pharmacological histone deacetylase (HDAC) inhibition restored Cldn5 expression and social interaction in mice. However, the mechanisms in humans were not elucidated (Dudek et al., 2020). These findings support a region-specific BBB mechanism in defined models but do not establish the universality of CLDN5 loss in depression.

Direct evidence in schizophrenia and bipolar disorder is not available. Serum HP or CLDN5 analysis did not reveal signal localization to the cerebral endothelium or quantify BBB permeability (Usta et al., 2021). Organoids derived from patients with schizophrenia exhibit altered endothelial-like cell proportions, angiogenesis-related transcription, and permeability phenotypes. However, the organoid vascular structures do not reproduce a perfused adult BBB (Stankovic et al., 2024). Studies using induced pluripotent stem cell-derived brain microvascular endothelial-like cells may identify disease-associated cellular phenotypes. However, differentiation fidelity, medication history, and donor heterogeneity limit causal inference (Lizano et al., 2023). Thus, endothelial aberrations may be components or consequences of broader inflammatory, metabolic, developmental, and treatment-related processes.

### 3.3 Sex-specific and brain region-specific neurovascular responses

Paired stress studies indicate that BBB responses vary according to sex, stress paradigm, and brain region. The chronic social defeat stress studies identified Cldn5 loss and permeability in the nucleus accumbens in male mice. However, studies with female mice revealed prominent prefrontal-cortical vascular and Cldn5 changes after subchronic variable stress (Menard et al., 2017; Dion-Albert et al., 2022). Region-specific Cldn5 reduction in the female prefrontal cortex was sufficient to increase anxiety-like and depression-like behaviors in the model. Human postmortem data from women and men support the relevance of CLDN5 alterations but do not define a reproducible sex-specific biomarker. Thus, the findings from one male or female model must not be extrapolated to all patients. Future studies must account for sex, hormonal status, brain region, medication exposure, and vascular comorbidity (Figure 3).

**Figure 3:**
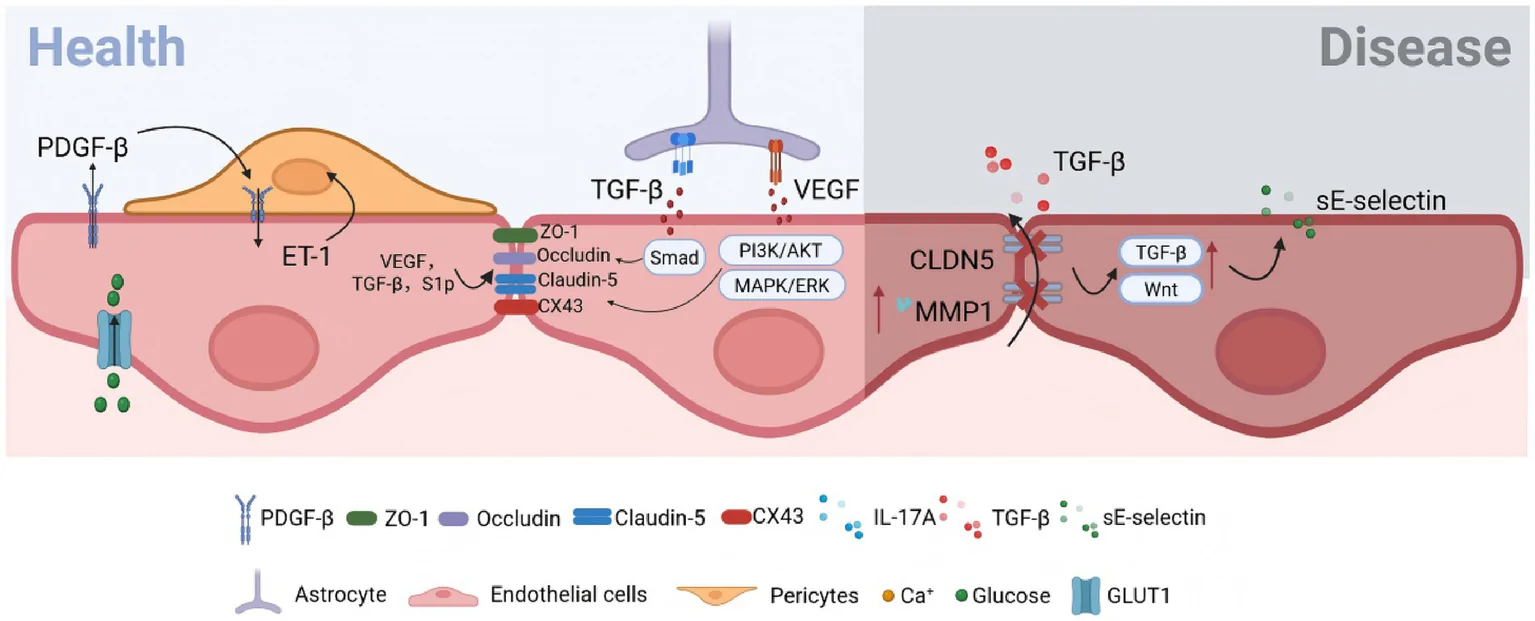
Molecular and cellular regulation of the brain endothelial barrier. Brain endothelial barrier properties are dependent on tight junction proteins (CLDN5, OCLN, F11R, and TRJP1), CDH5-based adherens junctions, the actin cytoskeleton, selective transporters, and the suppression of transcytosis. Pericytes, astrocytes, and basement membranes provide segment-dependent and context-dependent regulatory signals. Disease-associated changes should be distinguished based on the evidence source. Studies on human biomarkers and postmortem tissues provide associative data, whereas those on cell and animal models reveal specific mechanisms.

## 4 Brain endothelium in cerebral fluid and solute clearance pathways

Cerebral fluid and solute clearance involves the following anatomically and mechanistically distinct routes: perivascular CSF-interstitial fluid exchange, transport across the BBB, and drainage from CSF to meningeal lymphatic and venous outflow pathways. BECs are essential for maintaining vascular integrity and promoting BBB transport. However, conclusive evidence is not available for the role of BECs as central controllers of the glymphatic system. The term glymphatic is most appropriately reserved for a proposed astroglia-associated perivascular exchange pathway rather than as a synonym for all brain waste clearance pathways (Ahn et al., 2019; Rasmussen et al., 2018; Iliff et al., 2012). Imaging-based studies in prodromal neurological disease revealed the feasibility of assessing glymphatic-related function but did not establish a psychiatric mechanism (Chen et al., 2025).

Perivascular spaces, which are compartments surrounding penetrating vessels, are bound by vascular and glial basement membranes. The anatomy of perivascular spaces changes along the arterial-capillary-venous tree. Experimental tracer studies support CSF entry along periarterial routes, exchange with interstitial fluid in the parenchyma, and clearance along multiple downstream routes (Iliff et al., 2012) AQP4 in astrocytic endfeet is involved in this exchange in several animal models, although the reported magnitude and mechanism of convective flow vary. The original AQP1-AQP4 formulation is not retained because endothelial AQP1 has not been established as a necessary partner for glymphatic exchange. Similarly, BBB permeability is not equivalent to perivascular CSF movement. Opening endothelial junctions may promote plasma leakage without improving physiological CSF-interstitial fluid transport.

BBB-mediated efflux constitutes a distinct clearance mechanism. Endothelial LRP1 and ABCB1-selected ligands, including amyloid-beta, are transported from brain-facing compartments toward the blood. These components serve as substrate-specific transport systems rather than general-purpose waste pumps (Zlokovic et al., 2010). Meningeal lymphatic vessels drain CSF-associated fluid and immune components toward deep cervical lymph nodes (Ahn et al., 2019). These vessels are located outside the brain parenchyma and should not be conflated with cerebral veins or with endothelial transport across the BBB. Interactions among arterial pulsatility, sleep state, astrocytic endfeet, BBB transport, CSF outflow, and meningeal lymphatics are plausible but require separate measurements.

Currently, direct evidence for psychiatric symptoms arising from BEC regulation of glymphatic clearance is lacking. Sleep disturbance, vascular dysfunction, inflammation, and altered CSF dynamics frequently coexist in psychiatric illness. However, the co-occurrence of these conditions does not establish a BEC-glymphatic causal pathway. A defensible working hypothesis is that endothelial dysfunction could indirectly alter perivascular geometry, pulsatility, basement-membrane composition, or BBB transport, influencing cerebral clearance. This hypothesis must be tested with psychiatric cohorts or disease-relevant models to simultaneously examine endothelial function and perivascular tracer or imaging outcomes.

## 5 Translational opportunities and limitations of BEC-directed strategies

The therapeutic literature should be interpreted as an evidence hierarchy rather than as proof of an established BEC-targeted treatment class. The following four levels need to be distinguished: human endothelial associations, pharmacological treatments that incidentally engage endothelial pathways, preclinical interventions that improve barrier or vascular phenotypes, and interventions demonstrated to be endothelial-cell-specific. Clinical trials for a BEC-directed therapy have not been performed for a primary psychiatric disorder. Thus, BECs are candidate therapeutic interfaces and not clinically validated targets.

Human studies have provided only associative data. Analysis of postmortem major depressive disorder samples revealed that HDAC1 and CLDN5 expression varied according to diagnosis and antidepressant exposure. Causal HDAC inhibition and restoration of Cldn5 were demonstrated in the stress mouse model rather than in patients (Dudek et al., 2020). The reported association between antidepressant use, hippocampal angiogenesis, and progenitor cell proliferation was also based on postmortem tissue analysis and does not demonstrate the therapeutic activation of BECs (Boldrini et al., 2012). *In vitro* inhibition of endothelial serotonin uptake by paroxetine or fluoxetine and peripheral microvascular serotonergic aberrations indicate endothelial engagement. However, these findings do not demonstrate that endothelial modulation mediates clinical antidepressant efficacy (Brust et al., 2000; Greaney et al., 1985).

Preclinical studies provide testable strategies. Stabilizing Cldn5, limiting endothelial inflammatory signaling, or restoring regulated transcytosis may protect the BBB in selected stress models (Dudek et al., 2020; Menard et al., 2017). The FPR2 antagonist WRW4 improved BBB integrity and behavioral outcomes in socially isolated mice. However, FPR2 is prominent in capillary-associated microglia and other immune compartments. Thus, the intervention was not BEC-specific (Zheng et al., 2025). Ferulic acid improved endothelial and behavioral phenotypes in rats through a multi-organ signaling network without endothelial-selective manipulation (Xu et al., 2025). A study on acid sphingomyelinase inhibition and extracellular vesicles examined post-ischemic angiogenesis and stroke recovery and not as a treatment for a primary psychiatric disorder (Mohamud Yusuf et al., 2022). Older observations on monoamine oxidase and endothelial oxidative metabolism are mechanistically interesting but insufficient to support a psychiatric treatment claim (Richardson, 1993).

Translation will require both target specificity and vascular safety. Excessive junction opening could increase exposure to circulating toxins or immune cells. Indiscriminate barrier tightening could impair nutrient delivery, immune surveillance, or therapeutic drug penetration. Pro-angiogenic approaches may promote aberrant vascular remodeling, whereas the inhibition of inflammatory or adhesion pathways could alter host defense. As endothelial targets are systemically expressed, blood pressure, coagulation, renal, retinal, and peripheral vascular effects must be assessed. The most informative next steps are endothelial-specific or vascular segment-specific interventions, pharmacokinetic confirmation of brain endothelial exposure, comparison with neuronal and immune cell mechanisms, and prospective human studies using validated BBB or cerebrovascular biomarkers.

## 6 Conclusion

BECs contribute to psychiatric-relevant vascular biology through three mechanistically distinct domains. First, capillary ion and metabolite sensing, endothelial Ca^2+^ signaling, arterial gap junction conduction, and mechanical feedback shape NVC in a segment-specific manner. Second, junctional complexes, cytoskeletal anchoring, selective transporters, and suppression of transcytosis establish BBB integrity. Third, endothelial BBB transport operates alongside—but is anatomically distinct from—perivascular CSF exchange and meningeal lymphatic drainage. These distinctions prevent the broad attribution of all cerebral perfusion, barrier, and clearance aberrations to a single endothelial mechanism.

The psychiatric evidence is heterogeneous. Stress models support causal roles for selected endothelial pathways, especially sex-dependent and brain region-dependent changes in CLDN5. However, most human studies have provided only associative data and are influenced by medication exposure, systemic inflammation, metabolic disease, and vascular comorbidity. Serum markers do not localize pathology to the brain endothelium. Additionally, postmortem studies cannot establish temporal changes. Furthermore, patient-derived organoids do not fully reproduce a perfused BBB. Therefore, claims should specify the species, tissue, vascular segment, disease model, and level of causal inference.

Future studies should combine prospective human BBB or cerebrovascular measurements with endothelial-specific manipulation in disease-relevant models. The following domains must be prioritized: the validation of disease-specific and region-specific biomarkers, analysis according to sex, hormonal status, medication exposure, and vascular comorbidity, confirmation of brain endothelial target engagement, assessment of effects on blood pressure, coagulation, renal, retinal, and immune functions, and drug delivery strategies. BECs are promising therapeutic interfaces. However, current evidence supports cautious mechanism-guided development rather than the designation of BECs as clinically validated psychiatric targets.
